# Dog brain representations of human facial expressions: Encoding happiness and differentiating specific negative expressions

**DOI:** 10.1016/j.isci.2026.116900

**Published:** 2026-08-10

**Authors:** Raúl Hernández-Pérez, Luis Concha, Attila Andics, Rodolfo Bernal-Gamboa, Laura V. Cuaya

**Affiliations:** 1 Social, Cognitive and Affective Neuroscience Unit, Department of Cognition, Emotion, and Methods in Psychology, Faculty of Psychology, University of Vienna, Liebiggasse 5, 1010 Vienna, Austria; 2 Neuroethology of Communication Lab, Department of Ethology, Institute of Biology, Eötvös Loránd University, Pázmány Péter Sétány 1/C, 1117 Budapest, Hungary; 3 ELTE NAP Canine Brain Research Group, Pázmány Péter Sétány 1/C, 1117 Budapest, Hungary; 4 Department of Behavioral and Cognitive Neurobiology, Institute of Neurobiology, National Autonomous University of Mexico, Campus Juriquilla, Boulevard Juriquilla 3001, Querétaro 76230, México; 5 Faculty of Psychology, National Autonomous University of Mexico, Circuito Ciudad Universitaria, Mexico City 04510, México

**Keywords:** dogs, social perception, human facial expressions, affective neuroscience, functional magnetic resonance imaging, fMRI

## Abstract

Dogs can distinguish human facial expressions, particularly happiness, yet brain processes remain unclear. Using fMRI, we conducted two experiments in awake pet dogs. In Experiment 1 (*n* = 8), happy faces elicited a stronger response than neutral faces in a right temporal cluster extending to the caudate nucleus, including the rostral Sylvian gyrus. In Experiment 2 (*n* = 12), dogs viewed faces expressing happiness, anger, fear, or sadness. Using the Experiment 1 cluster as a region of interest, a machine-learning classifier distinguished happiness from each negative facial expression, but not between negative pairs, showing differential BOLD responsiveness to happy faces. Whole-brain representational similarity analyses revealed activity patterns differentiating angry vs. fearful faces (right mid ectosylvian and left splenial gyri) and sad vs. fearful faces (right rostral suprasylvian gyrus) but not angry vs. sad faces. This provides direct evidence that dog brains can distinguish between two negative facial expressions.

**Video abstract:**

## Highlights

• Happy human faces elicit right frontotemporal-caudate responses in dogs
• Dog brain response patterns distinguish happiness from negative facial expressions
• Whole-brain response patterns differentiate some pairs of negative facial expressions
• Dog brains represent human facial expressions beyond positive-negative valence

## Graphical abstract

**Figure:**
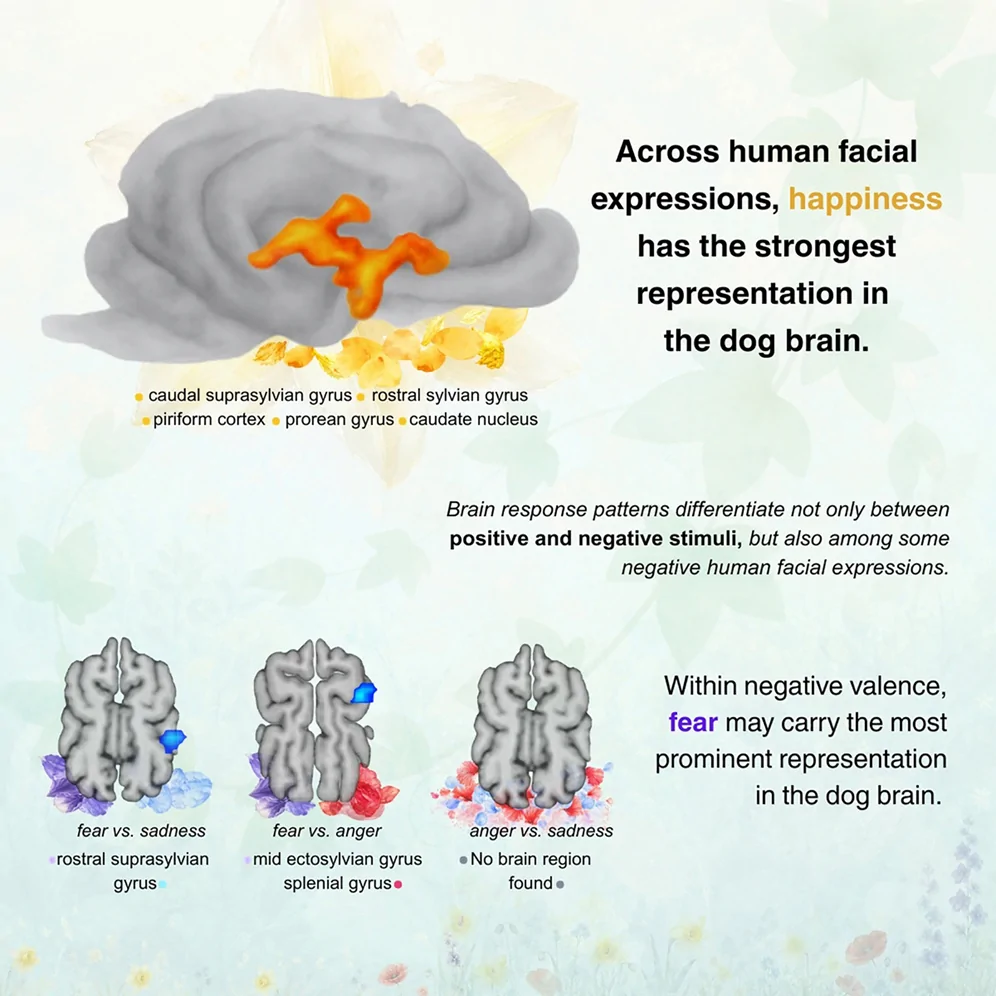

## Teaser

Canine behavior; Neuroscience; Cognitive neuroscience

## Introduction

Emotions serve as cues for interacting with the social world. Basic emotions, expressed through facial expressions that are universal in humans,^1^ are automatic physiological responses to certain stimuli and have high adaptive value. Face-based emotion recognition is widely observed in mammals, including sheep, monkeys, chimpanzees, and humans.^2^ Moreover, interpreting emotions in others is fundamental for communication between conspecifics across species.^3^^,^^4^ Some species possess communication abilities that extend beyond their own kind. For instance, dogs (*Canis familiaris*) have remarkable abilities to communicate with humans, including the ability to interpret human facial expressions, thereby enhancing their fitness in an anthropogenic environment.

Behavioral studies have shown that dogs are sensitive to emotional human faces, as evidenced by their longer visual examination of emotional faces compared to neutral faces.^5^ Dogs can discriminate between human faces expressing happiness and those expressing anger,^6^^,^^7^ fear,^8^ sadness,^9^ disgust,^10^ or neutrality^11^^,^^12^, as well as between angry^6^^,^^7^ and neutral faces.^12^ Furthermore, once dogs learned to differentiate a smiling face from a neutral one using photographs of their caregivers’ faces, they could generalize their learning to other humans of the same gender as their caregivers.^11^ Additionally, dogs and humans share some similarities in processing emotional human faces, such as presenting a bias to the left hemiface and spending more time looking at the left hemiface of a face with a positive valence.^13^ Moreover, an eye-tracking study^14^ found that dogs use a combination of information from the eyes, midface, and mouth (i.e., inner face) to process faces. However, when looking at a positive human face, dogs spend significantly more time looking at the eyes than at the mouth, suggesting that a positive human face elicits different processing in dogs. Along with dogs’ sensitivity to and discrimination of human facial expressions, there is evidence that dogs can interpret them and modulate their behavior accordingly. For instance, dogs tend to choose a box identified by a human through a happy facial expression over one related to disgust or fear, suggesting that dogs, to some degree, use emotional human expressions as references.^8^^,^^10^ Dogs’ ability to distinguish between emotional expressions in human faces may explain their remarkable behavioral flexibility in social contexts.^15^

Neuroimaging studies have demonstrated that the dog brain is sensitive to human facial expressions. One study that used visual event-related potentials (ERPs) found that the dog visual cortex displayed emotional expression-dependent effects at 130–170 ms, and the brain response successfully distinguished happy faces from scrambled faces.^16^ Using functional magnetic resonance imaging (fMRI), a study identified a region within the dog’s left temporal cortex that exhibited a stronger response to human faces compared to dog faces, and this response was parametrically modulated by the emotional valence displayed on human faces.^17^ In a separate fMRI study, a greater response was found in the caudate, hippocampus, and amygdala related to emotional faces compared to neutral faces; the caudate exhibited both a greater response to emotional faces and a parametric modulation by emotional valence.^18^ Another fMRI study^19^ found a greater response to happy human faces than to angry faces in the hippocampus, whereas the parahippocampal gyrus showed a greater response to angry human faces than to happy ones; both temporal regions were also sensitive to familiarity, showing a greater response to the faces of caregivers than to those of another familiar person.

Despite evidence ofdogs’ ability to perceive human facial expressions, some aspects remain unknown. Behavioral designs have tested two human facial expressions (one positive and one negative) at a time, so it is unclear if dogs only perceive a difference in valence (positive vs. negative). Although neuroimaging studies have suggested a role for familiarity (i.e., presenting faces of familiar people to dogs), it remains unknown how familiarity influences the brain processing of human facial expressions in dogs. Additionally, since no previous study had compared the brain response to human facial expressions of the same valence, it is unclear whether dogs’ brains only process the valence of human facial expressions.

Therefore, in this study, we aim to reveal the neural correlates of processing human facial expressions in dogs using fMRI. To rule out the influence of familiarity, we presented dogs with images of faces of unfamiliar people. To disentangle the effect of valence, we presented dogs with human faces expressing three negative expressions (sadness, anger, fear) and one positive expression (happiness) as well. We hypothesize that (1) processing of happy human faces involves the temporal cortex in dogs, (2) there exist neural signatures related to processing happy faces that make their discrimination from other human facial expressions possible, and (3) the dog brain can distinguish between human faces showing negative expressions as well. In Experiment 1, we identified brain regions that showed a bigger response to happy human faces than to neutral human faces. The aim of Experiment 2 was 2-fold: first, to reveal which human facial expressions are encoded in the functional localizer from Experiment 1, and second, to investigate the brain regions where patterns of activity generalize within the same type of negative expression while discriminating between different types of negative expression. Together, our two experiments provide neural evidence that dogs’ brain representations of human facial expressions extend beyond valence alone, revealing a happiness-sensitive frontotemporal-caudate cluster and distinct whole-brain patterns of activity that differentiate some specific negative human facial expressions, showing that dogs’ brains can distinguish between expressions with the same valence.

## Results

### Experiment 1: Happy faces elicit stronger right temporal-caudate responses than neutral faces in dogs

**Figure 1:**
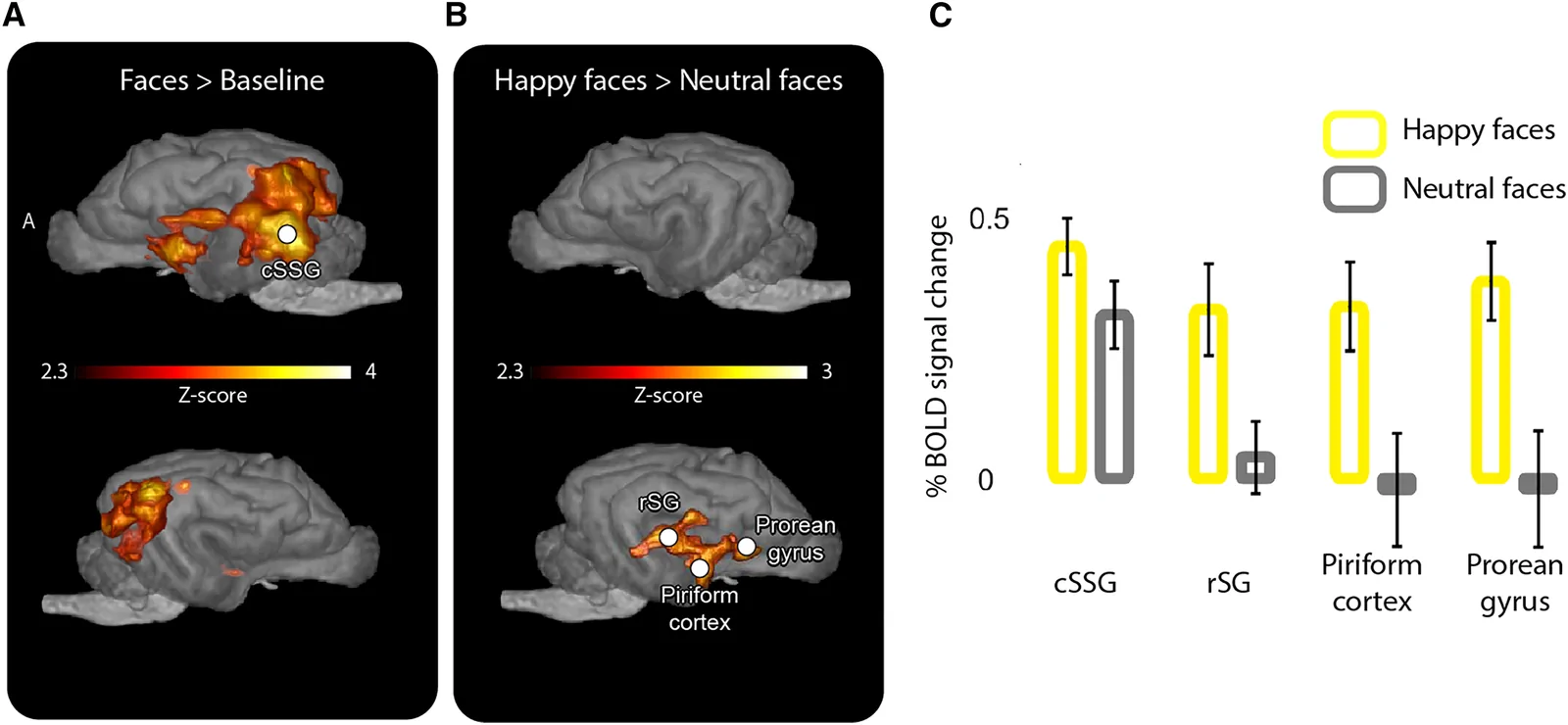
Functional localizer for happy human faces > neutral human faces (*n* = 8) (A and B) Volume rendering in lateral views showing resulting clusters overlaid on a dog template.^20^ (A) Faces > Baseline contrast; the white sphere indicates the voxel with the maximum z-score located in the occipital cortex. A = anterior; cSSG = caudal suprasylvian gyrus. (B) Happy faces > Neutral faces contrast; the white spheres indicate the three local maxima within the resulting cluster. rSG = rostral Sylvian gyrus. (C) The percentage BOLD signal change for happy and neutral faces within the spheres centered on the local maxima from both contrasts. rSG = rostral Sylvian gyrus. Vertical lines denote standard error of the mean.

To identify brain regions sensitive to human faces, we analyzed the cerebral activity resulting from viewing both happy and neutral faces using the contrast Faces > Baseline (Figure 1A). The activity related to viewing faces, regardless of their expression, included the bilateral occipital cortex and left temporal cortex.

To describe the dog’s brain response to happy human faces, we contrasted the BOLD activity between the two viewing conditions (i.e., Happy human faces > Neutral human faces), which resulted in a large cluster in the right temporal cortex that extended to the caudate nucleus (25,327 mm^3^; Figure 1B). Figure 1C shows the BOLD signal change from spheres of 5 mm radius centered at three local maxima of the resulting right-temporal cluster, located in the rostral Sylvian gyrus (rSG), prorean gyrus, and piriform cortex, and at the local maxima from the contrast Faces > Baseline located in the caudal suprasylvian gyrus.^20^ The coordinates, localization, and z-score of the local maxima are listed in Table 1. To test the lateralization, we created three spheres at the same locations as the three local maxima (Table 1) in the left hemisphere. We did not find any significant differences in the activity elicited by happy or neutral faces in the temporal regions of the left hemisphere (paired *t-*test, *p* > 0.05). The opposite contrast (neutral human faces > happy human faces) showed no significant difference.

**Table 1:** Local maxima resulting from the contrast Happy faces > Neutral faces

Z-score	x	y	z	Region
2.85	19	−10	5	right rostral Sylvian gyrus
2.82	11	11	1	right prorean gyrus
2.77	16	−2	−5	right piriform cortex

In summary, Experiment 1 provides fMRI evidence of the brain processing of happy human faces in dogs. As predicted, the results showed that the temporal cortex was involved in the processing of happy human faces. However, the results also revealed that other regions, such as the caudate nucleus, piriform cortex, and prorean gyrus, are involved in the processing of happy human faces.

### Experiment 2

The processing of emotional facial expressions does not occur in isolated brain regions, but rather through widespread networks.^21^^,^^22^^,^^23^ Thus, some brain regions may be involved in processing multiple facial expressions, making it challenging to study basic emotions using only univariate methods. Multivariate pattern analysis (MVPA) allows the decoding of information related to neural representations from distributed patterns in the brain. Using MVPA in humans, it has been possible to classify neural signatures associated with specific emotions and predict which emotion is being processed in the brain based on these patterns.^23^ In Experiment 2, we used MVPA to decode the perception of happiness, sadness, anger, and fear.

### Experiment 2A: The Experiment 1 localizer distinguishes happiness from negative expressions

**Figure 2:**
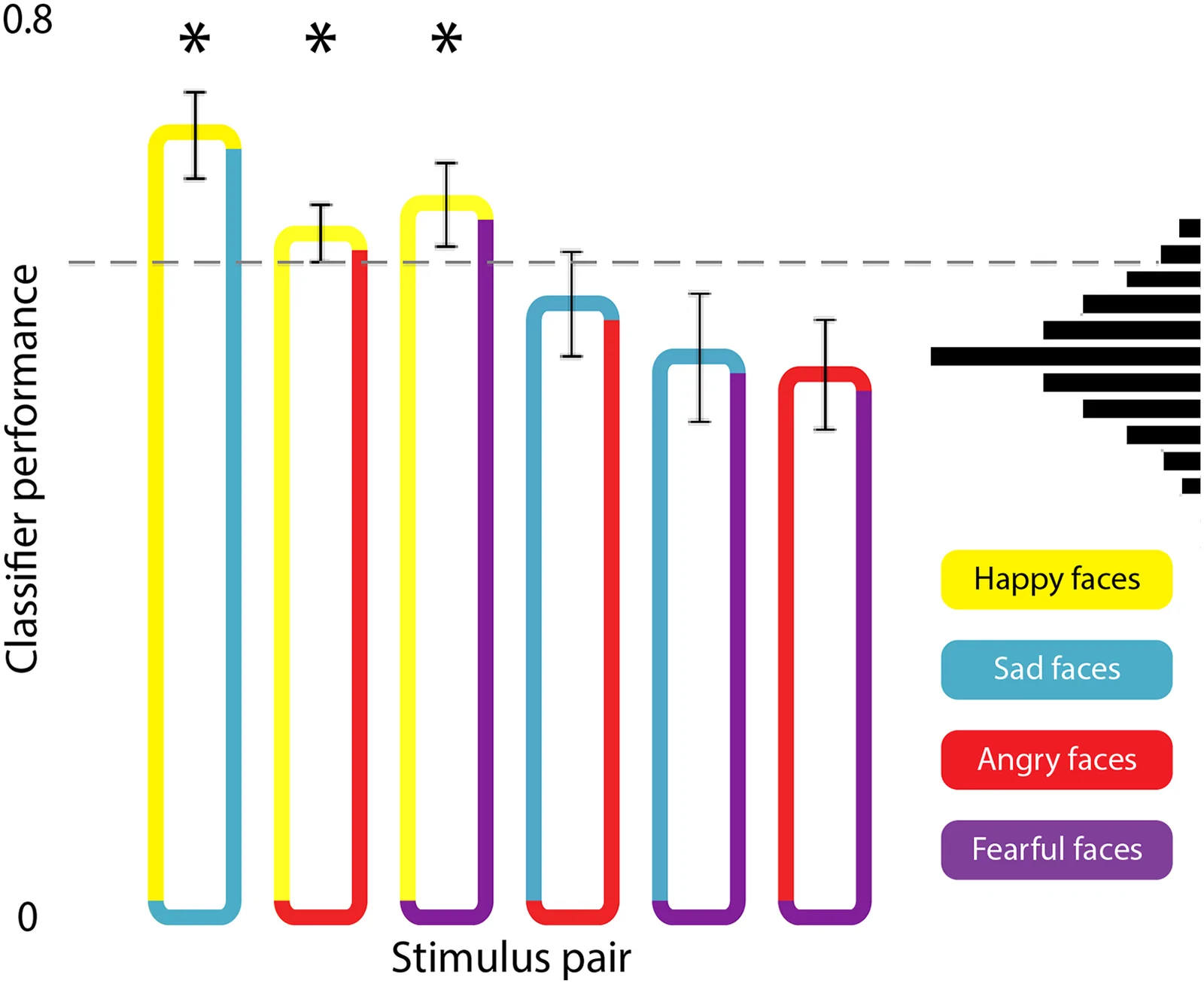
Classifier performance in discriminating between pairs of human facial expressions (*n* = 12) A linear support vector machine (LSVM) classifier was trained using the time series from voxels contained within the functional localizer identified in Experiment 1 as being responsive to happy human faces. Vertical lines denote the standard error. Asterisks indicate classifier performance significantly above chance as determined by permutation testing; ∗*p* < 0.05. The permutation-based null distribution is shown in black on the right. The dotted line represents the classification accuracy at *p* < 0.05.

Due to the design of Experiment 1, it is possible that the resulting cluster related to the processing of happy human faces responds to human facial expressions in general rather than happy human faces. To better understand whether the resulting cluster from Experiment 1 reflected preferential sensitivity to happy faces or broader sensitivity to human facial expressions, we presented twelve dogs with new images of human facial expressions while acquiring functional images. Because decoding was performed on independent acquisitions with non-overlapping stimuli relative to the Experiment 1 localizer, ROI definition and testing were statistically independent, avoiding circular analysis.^24^ Using only the time series derived from the functional localizer related to processing happy human faces (i.e., the result from Experiment 1; Figure 1B), we evaluated whether our classifier was able to discern each pair of human facial expressions. From these data, classification performance was above chance (*p* < 0.05) only when discriminating between happiness and any of the other three human facial expressions (Figure 2; classifier performance per pair: Happy vs. Sad faces = 0.698, range: 0.500–0.875; Happy vs. Angry faces = 0.609, range: 0.500–0.812; Happy vs. Fearful faces = 0.635, range: 0.438–0.812; Sad vs. Angry faces = 0.547, range: 0.312–0.812; Sad vs. Fearful faces = 0.500, range: 0.062–0.812; and Angry vs. Fearful faces = 0.484, range: 0.312–0.875). To assess whether the average classification performance was disproportionately driven by individual dogs, we evaluated the distribution of accuracies across the sample for each of the six binary comparisons. Using Tukey’s 1.5 × interquartile range criterion, we identified only two isolated outliers across the full set of dog-level classification accuracies. We then performed a leave-one-dog-out influence analysis, which showed that removing any single dog produced only minimal changes in mean accuracy, with a maximum absolute shift of 0.040. Together, these analyses indicate that the reported classifier performance was not driven by a single dog or by systematic individual outliers. Importantly, the region of interest derived from Experiment 1 was used as a single contiguous mask. Because MVPA decodes distributed neural representations, the classification analysis evaluated the combined pattern of all voxels within this mask rather than the contribution of specific anatomical subregions. Therefore, these results should not be interpreted as indicating that classifier performance was driven by any single subregion within the mask. Because fMRI data for Experiment 2 were collected at two different research sites, we conducted a supplementary analysis to examine whether scanning location influenced classifier performance by comparing the mean classification accuracy of dogs scanned at the first site (*n* = 4) with that of dogs scanned at the second site (*n* = 8). A Mann-Whitney U test showed no statistically significant difference in classification accuracy between sites (site 1: M = 0.604, standard deviation (SD) = 0.047; site 2: M = 0.566, SD = 0.042; U = 24.5, *p* = 0.172).

In summary, Experiment 2A results, using independent data, showed that the functional localizer found in Experiment 1 exhibited differential BOLD responsiveness to happy human faces rather than to human facial expressions in general.

### Experiment 2B: whole-brain representational patterns differentiate some negative human facial expressions of the same valence

With the results of Experiment 2A, we could not exclude the possibility that dogs merely processed valence, as we were only able to discriminate between positive (i.e., happiness) and negative (i.e., anger, sadness, and fear) human facial expressions. Experiment 2B was designed to test whether dog brains discriminated between negative human facial expressions. While the classifier in Experiment 2A was trained with the right temporal lobe data only (as this region was identified as being related to the processing of happy human faces), this experiment evaluated the dissimilarity between activity patterns derived from the entire brain.

**Figure 3:**
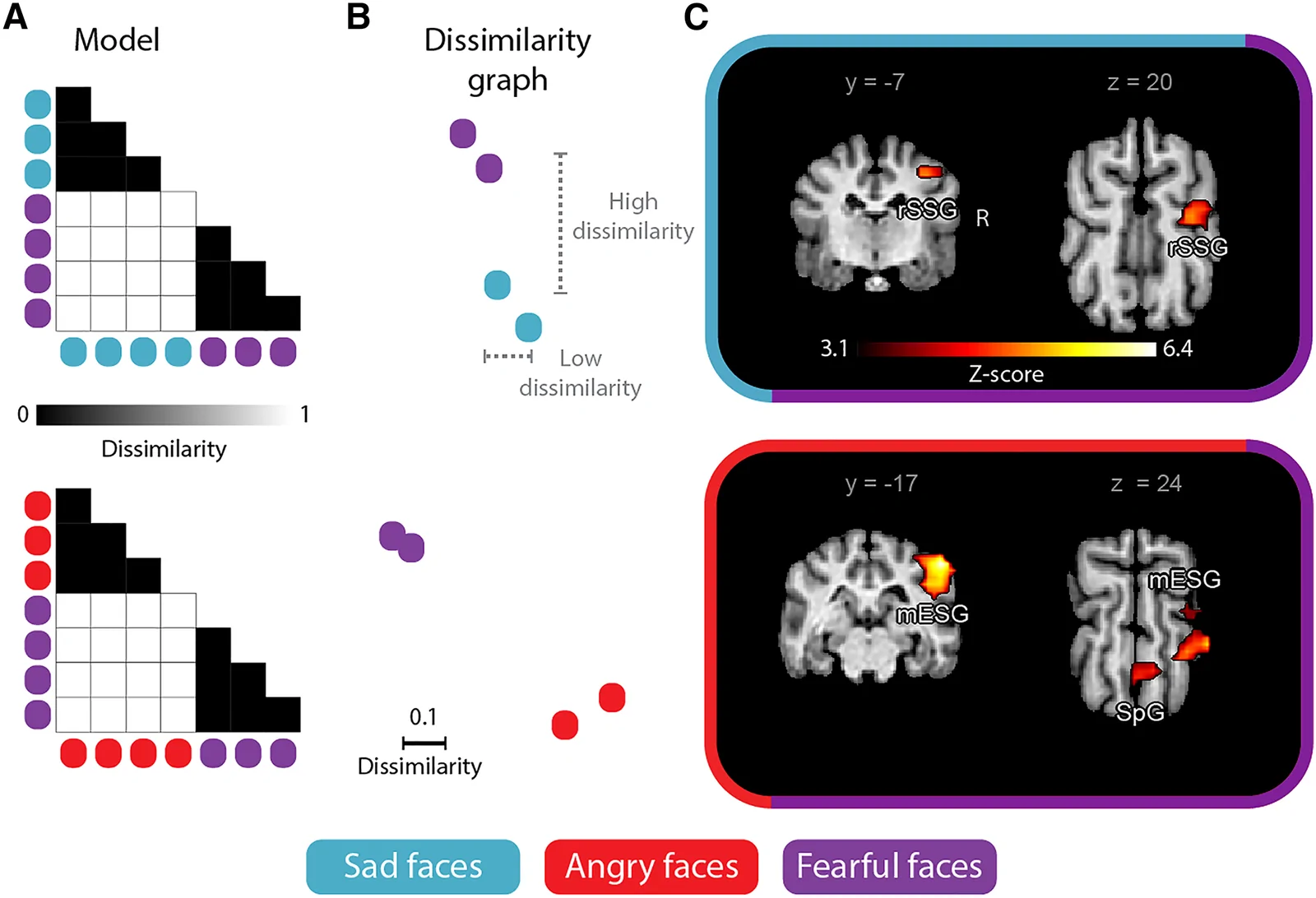
Brain regions distinguish between negative human facial expressions (*n* = 12) (A) The two models with significant results. Colored circles represent a stimulus block, and the color at the intersection is the predicted dissimilarity. Dissimilarity was measured by computing the Pearson’s correlation coefficient between the voxel activity patterns associated with each stimulus block and then subtracting the result from 1. The resulting value measures the dissimilarity between the different stimulus blocks, with a value of 0 indicating maximum similarity and a value of 1 indicating maximum dissimilarity. (B) Example of a multidimensional scaling arrangement of dissimilarities in two runs. Each circle represents a stimulus type in one run. The distance between circles represents the group-level dissimilarity calculated within a sphere (radius = 3 voxels) centered on the highest peak identified for each model; different-facial expression pairs have high dissimilarity, whereas same-facial expression pairs have low dissimilarity. (C) Brain regions with above-threshold correlation (z-score >3.1, cluster corrected at *p* < 0.05) using a searchlight approach (radius = 3 voxels) overlaid on a dog template.^20^ The sad faces vs. anger faces model yielded no significant results. R = right; rSSG = rostral suprasylvian gyrus; mESG = mid ectosylvian gyrus; SpG = splenial gyrus.

Using RSA, we tested whether cerebral patterns in the dog brain generalized between blocks of the same human facial expression, while discriminating across different human facial expressions. We determined which brain regions had a pattern of activity that correlated with that of a model that searched for similar activity to faces expressing the same facial expression but different activity to faces expressing different facial expressions. We found significant clusters related to sadness vs. fear (in the right rostral suprasylvian gyrus) and Anger vs. Fear (in the right mid ectosylvian gyrus (R mESG) and the left splenial gyrus), but not to sadness vs. anger (Table 2; Figure 3). To assess the persistence of these neural representations across runs, we conducted a stability analysis within the significant clusters identified by the RSA (i.e., the right rostral suprasylvian gyrus, the R mESG, and the left splenial gyrus). For each participant, we calculated the SD of the dissimilarity values across runs within these regions. Lower SD values in the observed data, compared to the permuted data, indicate that the dissimilarities are more consistent across runs. The results showed that, in all three regions, the observed data exhibited significantly lower SD values than those in the permuted data (*p* < 0.05).

**Table 2:** Brain regions distinguish between negative human facial expressions

Contrast	Voxels	Z-score	Regions	x	y	z
Sadness vs. fear	39	4.579	right rostral suprasylvian gyrus	15	−7	20
Anger vs. fear	237	6.427	right mid ectosylvian gyrus	21	−17	22
–	–	4.183	left splenial gyrus	−1	−27	24
Sadness vs. anger	n.s.	–	–	–	–	–

Threshold for reporting was z > 3.1, cluster corrected at *p* < 0.05. All peaks ≥ 8 mm apart are reported. n.s. = no significant clusters.

## Discussion

This study aimed to reveal the neural correlates of dog perception of happy human faces and to determine whether a unique pattern of brain activity is evoked by four basic human facial expressions (i.e., happiness, anger, sadness, and fear). Using whole-brain GLM analysis, we identified a cluster of activity centered in the temporal cortex and extending to the caudate nucleus, which was related to the processing of happy human faces (Figure 1B). Subsequently, we utilized MVPA with a linear support vector machine (LSVM) classifier to demonstrate that this cluster exhibited a preferential responsiveness to happy human faces rather than to human facial expressions in general, as it could differentiate happy human faces from those showing negative facial expressions (anger, sadness, and fear), but not between human faces showing two negative expressions (Figure 2). Additionally, employing whole-brain RSA, we discovered that the rostral suprasylvian gyrus exhibited patterns of activity that differentiated Sadness vs. Fear, while the mid ectosylvian gyrus and the splenial gyrus distinguished Anger vs. Fear. Our findings indicate that the dog brain can distinguish between positive and negative human facial expressions, and between some negative facial expressions. Our findings do not assert human-like semantic encoding of emotions in dogs. Rather, under stringent controls for low-level visual properties and HMAX-captured early-to-mid-level features, we show that dog brains exhibit different neural responses to different human facial expressions, such that patterns of activity (1) separate facial expressions by valence and (2) distinguish among some negative facial expressions. Importantly, our data do not identify which specific facial features drive these distinctions. The expression labels (i.e., happiness, anger, sadness, and fear) used here were defined in human-validated stimulus databases, and thus should be interpreted as descriptors of the stimuli rather than descriptors of dogs’ subjective experience. Claims are therefore restricted to brain representational separability, not to human-analog semantic categories. Importantly, representational separability in the RSA should not be interpreted as evidence that individual dogs would necessarily discriminate these facial-expression contrasts perceptually or behaviorally. To the best of our knowledge, this is the first evidence that dogs can distinguish between two human faces showing negative expressions. This suggests that the dog brain separates human facial expressions by valence and shows separability among some negative human facial contrasts, particularly those involving fear. These results provide insights into the neural mechanisms underlying dog perception of human facial expressions and may have implications for dog-human interactions.

### Positive human facial expressions

Our results revealed that the dog brain responds to happy human faces with a cluster located in the right hemisphere, centered in the temporal cortex (Figures 1 and 2). These findings are consistent with previous studies that found a stronger response in the dog temporal cortex when processing human faces compared to objects.^17^^,^^25^^,^^26^^,^^27^ Furthermore, an ERP study^16^ reported a distinct brain response in dogs toward happy human faces and neutral human faces in the dog visual cortex. Our study builds upon these findings by identifying that the strongest response to happy faces is located in the superior portion of the temporal lobe, specifically in the rSG. Auditory studies have suggested that the rSG is sensitive to emotional valence in human vocalizations and to language familiarity.^28^^,^^29^ Visual studies have shown a stronger response in the rSG to happy faces of a familiar person compared to strangers,^19^ and have identified the rSG as part of the dog action observation network.^30^ These findings support the idea that the rSG integrates multisensory social information and may serve a function analogous to the human lateral temporal pathway.^31^

In addition to the rSG, the cluster associated with the perception of happy human faces included the caudate nucleus, piriform cortex, and prorean gyrus. Dog fMRI studies have shown that the caudate nucleus is activated by representations of food reward^32^^,^^33^^,^^34^^,^^35^ and familiar scents.^36^ The caudate nucleus has also shown a stronger response to faces than to objects.^25^ Interestingly, a study^19^ found a greater response in the caudate nucleus to happy faces of the dog’s caregiver, a familiar person, and a stranger, as well as to angry faces of the caregiver compared to angry faces of a familiar person and a stranger. The authors suggested that the caudate nucleus is linked to the processing of rewarding stimuli (happy faces and the caregiver’s face). Similarly, another study^18^ found an increase in the caudate nucleus response to familiar and emotional human faces, with a parametric modulation by emotional valence. In a behavioral study, two groups of dogs were trained to identify either happy or angry faces; both groups of dogs learned to discriminate emotional human faces; however, the group trained to identify happy faces learned significantly faster.^7^ The authors suggested that dogs recognized an angry face as an aversive stimulus, while the inverse is plausible, with dogs showing accelerated learning due to the rewarding nature of happy human faces. Therefore, it is plausible that the perception of happy human faces not only functions as rewarding stimuli for dogs but also holds emotional significance, aligning with dogs’ social nature. Based on previous studies, we suggest that happy human faces, even those from strangers, are processed as a rewarding stimulus in the dog brain. The right piriform cortex has also been associated with processing happy human faces in dogs.^19^ Finally, the prorean gyrus, located in the dog’s prefrontal cortex, is part of a social action and interaction network associated with trainability^37^^,^^38^ and the development of pack structure in canids.^39^ The right prorean gyrus showed a stronger response to caregiver angry faces than to familiar angry faces,^19^ which could reflect a positive processing or familiarity effect. Importantly, a diffusion MRI study indicates that the prorean gyrus is anatomically connected to the rSG as part of a distributed frontotemporal network involved in socio-cognitive processing.^40^

Moreover, using the cerebral patterns from the cluster found in Experiment 1, a classifier could distinguish between pairs of human facial expressions when happiness was one of the facial expressions but not between two negative facial expressions (Figure 2). This result suggests that the rSG, caudate nucleus, piriform cortex, and prorean gyrus are preferentially involved in responses to happy faces relative to the negative facial expressions tested here. Some of the regions involved in processing happy faces are beyond the dog temporal cortex. Other studies have reported that the processing of human faces and emotional human faces is widespread (both spatially and temporally) in the dog brain.^16^^,^^19^^,^^41^^,^^42^ The model of the distributed human neural system for face perception^43^ proposes a hierarchical structure of face perception with a core system and an extended system. The core system includes the occipital and temporal cortices with the superior temporal sulcus (STS) related to the changeable aspects of faces (such as emotional expressions), in contrast to other face-sensitive regions such as the occipital cortex, which are not modulated by emotional facial expressions. The extended system includes cortices beyond the occipitotemporal visual extrastriate cortex. Our findings in dogs could be analogous to those in humans and compatible with the distributed model for face perception. The occipital lobe was involved in the early stages of face perception but was not involved in the coding of human facial expressions (Figure 1A). The superior temporal cortex in dogs could play a role similar to the STS in the processing of dynamic aspects of a happy human face. In contrast, the activity seen in the frontal regions (prorean gyrus) and caudate nucleus may suggest the participation of these structures as part of an extended system for face perception in dogs (Figure 1B). However, this cross-species mapping should be interpreted cautiously, as anatomical analogy or similar activation patterns alone are insufficient to support inferences about functional homology. Future studies should examine whether the dog brain can distinguish between pairs of positive human facial expressions to determine whether its ability to distinguish between facial expressions of the same valence also extends to positive valence facial expressions, and whether the dog brain shows a parametric response to facial expressions varying in valence, which could shed light on the mechanisms underlying emotion perception.

### Negative human facial expressions

The dog’s brain separates the presented human facial expressions by valence (positive vs. negative), and we found that the cerebral patterns could distinguish some pairs of negative human facial expressions (Figure 3). Our results showed that the patterns of activity from the cluster related to positive human facial expressions were unable to differentiate between pairs of negative human facial expressions, supporting our interpretation that these regions are primarily involved in processing faces expressing a positive human facial expression. Using a whole-brain approach, we found that patterns of activity in the right rostral suprasylvian gyrus could distinguish between sad and fearful faces, suggesting that this region may play a role in processing relevant social stimuli. Previous studies have shown that the suprasylvian gyrus exhibits a greater response to videos of familiar human social stimuli^19^ and human-dog interactions.^44^ In addition, we found that patterns of activity in the R mESG (part of the temporal cortex) and the splenial gyrus (part of the occipital cortex) can distinguish between angry and fearful faces. The R mESG may be involved in detecting potentially negative social stimuli; for example, a study found a bigger response in the R mESG to faces of strangers than to faces of familiar individuals.^19^ The splenial gyrus, which is part of the dog’s visual pathway,^19^^,^^41^^,^^42^^,^^44^^,^^45^ is also involved in processing animated stimuli (faces and bodies vs. objects)^42^ and emotional human faces, including angry faces.^19^ Similar to the behavioral evidence,^46^ we did not find any cerebral patterns that could distinguish between sad and angry faces. It is possible that differentiating between faces expressing positive vs. negative valence is relatively easy for the dog brain, whereas distinguishing between human facial expressions from the same valence may be more challenging. Because low-level features and HMAX-based early-to-mid-level visual features did not differ systematically within categories, negative human facial expression dissociations are unlikely to be driven by trivial visual regularities. An eye-tracking study found that when dogs viewed videos of humans expressing emotions, they primarily relied on body expressions for information, with facial expressions being secondary.^47^ This suggests that dogs may require additional cues beyond facial expressions, such as body expressions or information from other sensory modalities, to differentiate anger from sadness. Supporting this, when a caregiver expressed authentic sadness, their dogs obeyed less and kept a greater distance, indicating sensitivity to genuine human emotions expressed through multiple modalities.^48^

The asymmetric separability of fearful faces may be understood in terms of salience, arousal, and behavioral relevance across sensory modalities, rather than categorical emotion encoding. Regarding salience, an eye-tracking study^49^ revealed that dogs focused more on the nose of both fearful and neutral human faces, suggesting that they may inspect specific regions of fearful faces more closely. Moreover, a behavioral study showed that dogs attended to fearful faces but avoided angry faces,^12^ which may help explain the distinctiveness of fearful faces in our data. An arousal-based alternative is also plausible: fearful and angry human faces have been shown to elicit faster orienting responses and higher cardiac activity in dogs than lower-arousal expressions such as sadness.^50^ Converging auditory evidence points in the same direction: compared with sadness, fear vocalizations were associated with higher stress-related responses in dogs, suggesting that fear may elicit stronger reactions even among negative emotional cues.^51^ Fear may also have greater evolutionary and behavioral relevance: while adult dogs modulate their behavior in response to both human fear and happiness chemosignals, puppies respond distinctively only to fear chemosignals,^52^^,^^53^ suggesting that sensitivity to fear may be either biologically prepared or acquired earlier in development, whereas responses to other emotional chemosignals may depend more strongly on socialization. Viewed in this light, our results raise the possibility that the dog brain processes fear in relation to anger and sadness through partly different neural mechanisms, as these contrasts were represented in different brain regions.

### Lateralization

Our results indicate a tendency for right-hemisphere brain lateralization to process human facial expressions. In Experiment 1, all brain activity related to happy faces was observed in the right hemisphere. However, we believe that this tendency was an effect of the statistical threshold used, as activity related to happy faces was also present in the left hemisphere. In Experiment 2B, cerebral patterns distinguishing negative human facial expressions were primarily located in the right hemisphere, although the cluster that distinguished Angry vs. Fearful faces included a part of the left splenial gyrus. Our findings align with an interesting study that found a left-head-turn bias (i.e., right hemisphere preferential processing) in dogs when viewing human faces expressing anger, fear, and happiness.^50^ Dogs also exhibited a left gaze bias toward emotional (positive and negative) human faces in an eye-tracking study,^46^ and turned their heads left more often toward visual human emotions than toward dog emotions.^47^ Our results support the right-hemisphere theory,^54^ which suggests that the right hemisphere processes all emotional information, regardless of its valence. In contrast, the valence theory^55^^,^^56^ posits that the left hemisphere is involved in processing positive emotions, whereas the right hemisphere is involved in processing negative emotions. Although our results do not support the valence theory, behavioral evidence across sensory modalities and motor responses (tail-wagging movements) in dogs suggests otherwise.^57^ It is possible that lateralized emotional processing in dogs depends on the species presented as stimuli and on the sensory modality.

### Distributed neural representations of emotion

Within a broader comparative framework, our combination of ROI-based decoding and whole-brain RSA is consistent with recent multivariate and representational approaches suggesting that socio-emotional processing relies on distributed neural patterns. In humans, emotion information can be decoded from whole-brain functional connectivity patterns extending beyond classical emotion-processing regions,^58^ visually evoked emotion representations appear high-dimensional and distributed across areas,^59^ and visual emotion perception involves recurrent temporo-occipital interactions.^60^ Comparative findings in non-human species point in the same direction, showing hierarchical and partially segregated processing of facial expression and identity across visual pathways in macaques,^61^ broadly tuned body-selective networks for affective body language,^62^ and emotion-related facial patterns linked to insular cortex activity in mice.^63^ Together, these findings suggest that emotion-related states across species are best approached as distributed, multisystem processes rather than as functions of single regions.^64^

A strong point of this study is that the dogs were presented with unfamiliar faces of both genders. Previous research has shown that dogs are more responsive to emotionally charged information expressed by their caregiver than by a stranger.^8^ Additionally, dogs have difficulty generalizing the discrimination of happiness on faces of the opposite gender to their caregivers.^11^ Although using faces from their caregivers may have improved the accuracy of the classifier in Experiment 2, we aimed for a more general identification of different human facial expressions.

In conclusion, our findings provide insight into the remarkable sensitivity of the dog brain to human facial expressions. Experiment 1 provides evidence that dog brains can distinguish between happy and neutral faces, as indicated by the broad brain activity observed in response to happy faces. Along with these results, Experiment 2A demonstrated that the cerebral patterns from the regions identified in Experiment 1 distinguished between happy faces and three negative human facial expressions, but not between two negative human facial expressions. In Experiment 2B, a whole-brain analysis revealed brain patterns that distinguish between pairs of negative human facial expressions (Angry vs. Fearful faces and Sad vs. Fearful faces), although we did not find evidence for different brain representations for Sad vs. Angry faces. Our results reveal for the first time that the canine brain possesses a representation of human facial expressions that goes beyond simple valence classification. Overall, our study highlights the significance of human social cues for dogs, as evidenced by the dog brain’s sensitivity to human facial expressions, and underscores the importance of human social information in shaping dogs’ social cognition.

### Limitations of the study

Our study has several limitations. Behavioral studies on emotion perception in dogs have demonstrated differences across breeds^65^^,^^66^ (although see Pongrácz et al.^67^). Therefore, we are cautious in generalizing our findings beyond the border collies that comprised the majority of our sample. Border collies have been shown to be highly skilled at following social cues,^68^ spend more time looking at humans, and are more willing to interact with them.^69^ Therefore, it is possible that border collies have a greater ability to interpret human facial expressions, and future studies are needed to determine whether there are behavioral and cerebral differences in the perception of human facial expressions across breeds. Additionally, the BOLD signal is inherently noisy in both humans and dogs, but the use of coils tailored to the anatomy of dogs may be necessary to reduce noise and improve data quality.^70^^,^^71^^,^^72^

Another limitation of our study is that we presented static facial expressions, as dynamic face stimuli may elicit stronger responses and increase ecological validity.^19^^,^^27^ Indeed, processing images of human facial expressions may be more challenging for dogs in a laboratory setting than in their everyday lives, where they primarily encounter dynamic social signals. For example, an eye-tracking study revealed that, for dogs, human social images require more demanding and detailed analysis than non-social or dog images.^73^ Our findings for positive facial expressions are broadly consistent with findings using dynamic positive facial expressions,^19^ suggesting that this effect may not be restricted to static stimuli. Regarding the use of 2D stimuli, dogs can discriminate people based on their faces,^74^ even from photographs,^75^ and an fMRI study found no brain regions that discriminated between 2D and 3D objects.^76^ In this context, a dog fMRI study found a temporo-occipital response to videos of faces and live faces^26^; however, live faces elicited a stronger response than videos of faces, only in the ectosylvian fissure.^27^ Future studies should therefore aim to increase ecological validity in line with recent recommendations to adopt a more dog-centric perspective,^77^ by testing additional conditions, such as non-face emotional stimuli, multimodal stimuli, and dynamic facial expressions, to determine whether the neural patterns reported here extend to more naturalistic emotional signals.

We also acknowledge the limitations regarding the sample size (*n* = 12) in the context of machine learning classification, which can increase the risk of overfitting in multivariate analyses. To mitigate the risk of overfitting and strengthen the validity of our results, we maximized pattern instances per subject, used out-of-run cross-validation, and relied on permutation-based inference at both subject and group levels. Specifically, we prioritized data density per subject by acquiring ten functional runs for each dog, ensuring a substantial number of volumes for robust pattern estimation. This within-subject depth was coupled with a multivariate pipeline where we employed a leave-one-out cross-validation scheme and determined statistical significance using permutation testing (*N* = 100,000) to generate empirical null distributions. This approach aligns with recent recommendations for statistical inference on representational geometries to prevent overfitting and ensure that conclusions generalize correctly.^78^ Nevertheless, despite these mitigation strategies, our sample size (*n* = 12) implies that a residual risk of overfitting cannot be fully ruled out, and the present findings should therefore be carefully interpreted. We acquired data from two sites in Experiment 2, with some differences in the acquisition parameters and hardware configurations. While such hardware disparities, specifically the use of different coil arrays, can influence the overall sensitivity and baseline signal-to-noise ratio of the BOLD signal, our primary analyses (Machine Learning Classification and RSA) were based on intra-participant measurements. Because classifiers were trained and tested on data originating from the same scanner, and dissimilarity matrices relied on relative correlation distances between conditions rather than absolute signal intensities, site-specific global differences were unlikely to have substantially biased the underlying neural patterns. In addition, a supplementary analysis showed no statistically significant difference in classification accuracy between sites. Although this analysis does not rule out subtle site-related effects, it does not provide evidence that classifier performance differed as a function of acquisition site. Another potential limitation of this study is our assumption that dogs and humans have a similar canonical HRF. A methodological study showed an increase in model fit and fMRI detection power in the dog visual cortex using a tailored dog HRF.^45^ Future dog fMRI visual studies may benefit from using live faces and a tailored dog HRF.^31^ As recommended by Correia-Caeiro et al.,^77^ it is necessary to shift focus from an anthropocentric perspective to a dog-centric perspective when studying social perception in dogs. This can be achieved by presenting emotional body stimuli, exploring a wider range of emotions beyond stereotypical human emotions, and incorporating multimodal stimuli whenever possible.

## Resource availability

### Lead contact

Further information and requests for resources should be directed to and will be fulfilled by the lead contact, Raúl Hernández-Pérez (raulh87@univie.ac.at).

### Materials availability

This study did not generate new unique reagents.

### Data and code availability

The data associated with this manuscript have been deposited at Zenodo, and the custom preprocessing and analysis code used in this study is available through the Dog Brain Toolkit on GitHub. Both resources are publicly available, and their DOIs are listed in the key resources table. Any additional information required to reanalyze the data reported in this work is available from the lead contact upon request.

## Acknowledgments

We would like to express our sincere gratitude to our participants and their caregivers for their invaluable cooperation, patience, and friendship throughout this study. We thank Erick Pasaye, Juan Ortiz, and Rita Báji for their support during image acquisition. We would also like to acknowledge the National Laboratory for Magnetic Resonance Imaging, UNAM, México, and the Department of Neuroradiology, Medical Imaging Centre, Semmelweis University, Budapest, for their assistance. We are deeply grateful to Azalea Reyes and Fernando Barrios for their thoughtful comments and to Magdalena Boch for her unwavering support and encouragement. Finally, we thank reviewers #4 and #5 for their careful review and constructive comments.

This research was funded in whole or in part by the 10.13039/501100002428Austrian Science Fund (10.13039/501100002428FWF): 10.55776/ESP602. For open access purposes, the author has applied a CC BY public copyright license to any author accepted manuscript version arising from this submission. This research was also funded by the 10.13039/501100000781European Research Council (ERC) under the 10.13039/501100007601European Union’s Horizon 2020 research and innovation program (grant no. 950159), the 10.13039/501100003825Hungarian Academy of Sciences via a grant to the MTA-ELTE Lendület Neuroethology of Communication Research Group (grant no. LP2017-13/2017), and the National Brain Programme 3.0 (NAP2022-I-3/2022). 10.13039/100020584R.H.-P. and L.V.C. were also funded by the 10.13039/501100003141Mexican National Council for Humanities, Science and Technology (10.13039/501100003141Conahcyt, 409258 and 407590, respectively). L.C. acknowledges support from 10.13039/501100003141Conahcyt (181508, 1782), from 10.13039/501100005739UNAM-DGAPA (IB201712, IG200117, IN204720), and from UNAM-PAPIIT (IT200626-3).

## Author contributions

R.H.-P.: conceptualization, conceived and designed the study, performed research, analyses, software, visualization, drafted the manuscript, and reviewed and edited the manuscript. L.C.: conceptualization, conceived and designed the study, drafted the manuscript, reviewed and edited the manuscript, and funding acquisition. A.A.: drafted the manuscript, reviewed and edited the manuscript, and funding acquisition. R.B.-G.: conceptualization and reviewed and edited the manuscript. L.V.C.: conceptualization, conceived and designed the study, performed research, analyses, drafted the manuscript, and reviewed and edited the manuscript.

## Declaration of interests

The authors declare no competing interests.

## STAR★Methods

### Key resources table

**Table:** 

REAGENT or RESOURCE	SOURCE	IDENTIFIER
**Deposited data**		

Experiments 1 and 2: fMRI raw data	This paper	Zenodo: https://doi.org/10.5281/zenodo.18475405
Experiment 1: GLM results	This paper	Zenodo: https://doi.org/10.5281/zenodo.18475405
Experiment 2: RSA searchlight group maps	This paper	Zenodo: https://doi.org/10.5281/zenodo.18475405
Experiment 2: Individual similarity maps	This paper	Zenodo: https://doi.org/10.5281/zenodo.18475405

**Experimental models: Organisms/strains**		

Dogs (*Canis familiaris*)	Private caregivers	N/A

**Software and algorithms**		

PsychoPy2 v 1.90	Peirce^79^	https://psychopy.org/
FSL (FMRIB Software Library)	Jenkinson et al.^80^	https://fsl.fmrib.ox.ac.uk/fsl/docs/#/
3D Slicer v 4.10	Kikinis et al.^81^	https://www.slicer.org/
PyMVPA v 2.6.5	Hanke et al.^82^	http://www.pymvpa.org/
LibSVM v 3.25	Chang and Lin^83^	https://www.csie.ntu.edu.tw/∼cjlin/libsvm/
MATLAB (2018b and 2023b)	MathWorks	https://www.mathworks.com/products/matlab.html
Python v3.1	Python Software Foundation	http://python.org/
Custom code for preprocessing and analyzing dog fMRI data through the Dog Brain Toolkit	This paper Hernández-Pérez^84^	GitHub: https://github.com/rhernandez00/dog_brain_toolkit

**Other**		

Stimuli presented in Experiment 1	AR Face Database^85^	http://www2.ece.ohio-state.edu/∼aleix/ARdatabase.html
Stimuli presented in Experiment 2	Karolinska Directed Emotional Faces database^86^	https://kdef.se/home/aboutKDEF

### Experimental model and study participant details

In Experiment 1, eight healthy family dogs (*Canis familiaris*), all from medium-sized breeds, including six border collies, one labrador, and one golden retriever, participated. Their ages ranged from 18 to 53 months, and the sample included four neutered males, one neutered female, and three unneutered females. All dogs living with human families were well socialized with humans and other dogs. Of the eight dogs included in this study, seven had previously participated in an fMRI experiment and had been trained to remain still inside a scanner in the sphinx position while watching images projected during fMRI acquisition. Those dogs did not require any additional training. The new participant (Molly) was trained a year after the other seven dogs using the same procedure.^25^ The procedures followed the Association for the Study of Animal Behavior (ASAB) guidelines, and the Bioethics Committee of the Institute of Neurobiology of the National Autonomous University of Mexico (UNAM) approved the study (26RM). Informed consent was obtained from the primary caregiver of each dog. All caregivers volunteered to participate in the study and did not receive any monetary compensation. The dogs were allowed to leave the sessions at any time. During the study, the dogs lived with their human families without changes in their routine, except for the training and imaging protocols described herein.

In Experiment 2, twelve healthy family dogs from three different breeds participated: one mixed breed, three golden retrievers, and eight border collies. Six dogs participated in Experiment 1. Their ages ranged from 26 to 142 months, and the sample included five males. The same guidelines as those in Experiment 1 were followed. Four dogs were scanned at the National Laboratory for Magnetic Resonance Imaging at the Institute of Neurobiology of the UNAM and eight dogs were scanned at the Department of Neuroradiology, Medical Imaging Centre, Semmelweis University. All procedures were conducted following the ASAB guidelines and approved by the Bioethics Committee of the Institute of Neurobiology of UNAM (26RM) in Mexico and by the National Animal Experimentation Ethics Committee (PEI/001/1490-4/2015) in Hungary. The primary caregiver of each dog gave informed consent.

### Method details

#### Experiment 1

##### Design and stimuli

We used a block design and presented images of human faces as stimuli. There were two types of blocks: faces with neutral and happy expressions. Each block was presented for 7 s, with an inter-trial period of 12.25 s used to estimate baseline activity (during which they observed a white screen with a small cross). Each fMRI acquisition (run) had a duration of 192.5 s and included five blocks of happy faces and five blocks of neutral faces. The blocks were presented in a pseudo-randomized order, and the same type of block was never presented three consecutive times. In total, each dog underwent five runs (each run had a different order of blocks). The dogs experienced only one to three runs with rest periods between them in each imaging session, and different sessions occurred on different days. We used short runs to maximize the attention of dogs to the stimuli. Thus, fMRI acquisition was completed over two to three non-consecutive days for each dog. PsychoPy2^79^ controlled the stimuli presentation. The stimuli were projected onto a screen in front of the dogs at 1.5 m. The lights in the MRI suite were turned off during the paradigm presentation and an experimenter was present in the MRI room to ensure the dog’s compliance during acquisitions.

Each block consisted of four different photographs of faces, all of which were extracted from the AR Face Database^85^; therefore, all stimuli were unfamiliar to the dogs. We presented the same individuals with both neutral and happy expressions, ensuring that each image was presented only once during each run to prevent habituation. In order to have stimuli as natural as possible, all images were in color, presented in a frontal view, and the size of the projected images was similar to that of a real face (15 cm × 20 cm), as this size has been used in other studies.^11^^,^^25^^,^^87^ An independent two-sample t-test showed no differences in low-level visual properties between happy and neutral female faces: hue t(98) = -0.069, *p* = 0.945; saturation t(98) = 0.158, *p* = 0.875; brightness t(98) = -0.240, *p* = 0.811; contrast t(98) = -0.190, *p* = 0.850; nor between happy and neutral male faces: hue t(98) = -0.210, *p* = 0.834; saturation t(98) = 0.133, *p* = 0.895; brightness t(98) = -0.323, *p* = 0.748; contrast t(98) = -0.330, *p* = 0.742. The gender of the faces presented to each dog was determined by the primary caregiver’s gender, with four dogs (one male and three females) seeing female faces and the other four dogs seeing male faces. We decided to show a specific gender for each dog based on a previous study that showed that dogs had lower accuracy in a behavioral task when presented with an unfamiliar face of someone of the opposite gender to their primary caregiver.^11^

##### Data acquisition

All images for Experiment 1 were acquired at the National Laboratory for Magnetic Resonance Imaging at the Institute of Neurobiology of UNAM. We used a 3 T Philips Achieva TX scanner and a two-channel SENSE Flex Small coil attached to the dogs with Velcro straps. To help the dogs maintain their sphinx position, we used a chin rest to support their heads. The dogs wore earmuffs for noise protection during scanning. The dogs were awake and unrestrained during all sessions and could leave at any time.

For anatomical reference, we also acquired a standard T1-weighted structural image with a turbo spin echo sequence with a resolution of 1 × 1 × 1 mm^3^, covering the whole brain with 75 slices. The whole-brain blood-oxygen-level-dependent (BOLD) images were acquired using a gradient-echo echo-planar imaging (EPI) sequence (28 coronal slices, 3 mm thickness, no gap; TR = 1.75 s; TE = 30 ms; flip angle = 90°; FOV = 224 × 240 mm^2^; acquisition matrix 112 × 120; spatial resolution 2 × 2 × 3 mm^3^; 110 volumes and 5 dummy scans).

##### Quantification and statistical analysis

The brain was extracted from each run using manual segmentation. All functional analyses were performed using FSL^80^ version 4.19, together with the Dog Brain Toolkit, a custom toolkit for preprocessing and analyzing dog fMRI data.^84^ Images were preprocessed for temporal correction, and spatial smoothing was performed using a Gaussian kernel with FWHM = 5 mm. Motion correction was performed using MCFLIRT, and any runs showing head rotation greater than 1° or translation greater than 3 mm were discarded (less than 10% of runs discarded; if a run was discarded, a new acquisition was obtained until each dog had five good-quality runs). Functional images were spatially normalized to the anatomical image of each dog and to a digital atlas of the dog's brain.^88^

We used a General Linear Model for the statistical analyses, including the stimulus vectors of happy and neutral faces as regressors. At the first level, each run was analyzed individually. Regressors were convolved with the canonical hemodynamic response function (HRF), modeled as a gamma function. A fixed-effects analysis was used to analyze the five runs of each participant. A random-effects analysis was used to study the common activations among the eight participants. To determine the cerebral regions involved in the perception of happy faces, we analyzed the contrast Happy faces vs. Neutral faces in the entire brain. The resulting statistical parametric maps were corrected for multiple comparisons using random field theory^89^ cluster-forming threshold z > 2.3 and p_cluster_ < 0.05. A sphere of 5 mm radius was created around the voxel of each of the three local maxima resulting from the contrast Happy faces > Neutral faces to extract the BOLD signal change.

#### Experiment 2

##### Design and stimuli

To avoid a familiarity effect, we presented the faces of human strangers to the dogs. We chose to use only four basic human facial expressions (happiness, sadness, fear, and anger) because even for humans it is difficult to discriminate between fear and surprise and between anger and disgust.^90^ The stimuli used were human faces expressing happiness, sadness, fear, and anger extracted from the Karolinska Directed Emotional Faces database.^86^ Note that Experiment 1 used AR Face Database images, whereas Experiment 2 used KDEF images, ensuring stimulus-set independence across experiments. One-way ANOVA showed no differences in low-level visual properties among the four human facial expressions (happiness, sadness, fear, and anger) with respect to hue (F(3, 316) = 0.572, *p* = 0.634), saturation (F(3, 316) = 2.290, *p* = 0.078), brightness (F(3, 316) = 1.972, *p* = 0.118), and contrast (F(3, 316) = 0.600, *p* = 0.629). To rule out the possibility that similarities in low-to-mid-level shape features among images within the same group could explain the classification accuracy, we used a computational model, HMAX,^91^ to simulate the combined response of neurons in the early visual cortex to image structure. Using this model, we determined the expected response of the early visual cortex to the presented images, calculated the dissimilarity (measured as the correlation distance) between the response vectors of the model’s C1 units, and compared the within- and between-group dissimilarities for each human facial expression (Figure S1). An independent two-sample t-test confirmed no significant differences between the two groups (*p* > 0.05). Thus, neither low-level feature distributions nor systematic differences in early-to-mid-level visual features captured by HMAX could account for classification/RSA effects.

We used a block design (each block was presented for 7 s and consisted of four different images of the same human facial expression, followed by a baseline of 12.25 s) in each run. A human facial expression block was presented twice in a pseudo-random order, and all faces in a block belonged to a single gender, which was switched between scans. The total duration per run was 166.25 s, and each dog experienced ten runs in total. The projection and dogs' positions were the same as in Experiment 1.

##### Data acquisition

For four dogs, we used the same procedure as in Experiment 1, except that each run consisted of 95 volumes. For the other eight dogs, we followed the same procedure but used a 3 T Philips Ingenia scanner with an eight-channel dStream Pediatric Torso coil. The whole-brain BOLD images were acquired using a gradient-echo echo-planar imaging (EPI) sequence (28 coronal slices, 3 mm thickness, no gap; TR = 1.75 s; TE = 30 ms; flip angle = 90°; FOV = 224 × 199 mm^2^; acquisition matrix 112 × 98; spatial resolution 2 × 2 × 3 mm^3^; 95 volumes and 5 dummy scans).

##### Quantification and statistical analysis

Preprocessing was performed using FSL^80^ and custom scripts available through the Dog Brain Toolkit.^84^ Runs exhibiting motion greater than 1° of head rotation or 3 mm of translation were discarded. Six runs were discarded. To ensure an equal number of runs per participant, the first eight runs that met the movement threshold per dog were retained. The mean frame-wise displacement^92^^,^^93^ was 0.136 mm across all dogs and runs, and the proportion of volumes with frame-wise displacement (FD) greater than 1 mm was 0.06%. All functional images were corrected for slice timing, high-pass filtered, and reoriented to match a dog template.^20^ All runs were skull-stripped by drawing a binary mask over the brain. For each dog, all images were aligned to the first volume of the first run using FLIRT. We then created a mean functional image by averaging all volumes using FSLUTILS. The mean functional image was manually co-registered to the dog’s structural image using 3D Slicer.^81^ The resulting image was manually co-registered to the dog template to create a normalized image. We used FLIRT to calculate a transformation matrix from the mean functional image to the normalized image, which was then applied to each run. The images were smoothed using a Gaussian kernel (5 mm FWHM). Beta estimates for individual runs were calculated using FEAT (FMRI Expert Analysis Tool) version 6.00, performing a General Linear Model. We used each human facial expression as a single regressor, convolving the time series of the regressors with the HRF. Multivariate analysis was performed using the PyMVPA software package^82^ and custom MATLAB scripts available through the Dog Brain Toolkit.^84^ For classification, we followed a leave-one-out cross-validation scheme, in which the runs were split into two sets: a training set comprising seven runs and a test set comprising the remaining run. This leave-one-run-out scheme ensured out-of-run generalization within dogs; importantly, Experiment 2A used stimuli and acquisitions independent from Experiment 1, limiting ROI circularity. Group-level significance was then assessed using permutation-based MVPA inference by resampling subject-level null distributions to form a group-level null for the mean accuracy, following Stelzer et al.^94^ We trained a linear support vector machine (LSVM) classifier (implemented in LibSVM^83^) using the training set and tested it using the test set, repeating the procedure using each run as a test set and the others as a training set. Only a pair of categories was used for a given training and testing fold, and the classifier performed a 2-way classification in which only classifications that matched the human facial expression were considered correct. To obtain the distribution of our data under a random classification condition, we conducted a permutation test in which we randomly swapped the block labels, trained and tested the classifier, and repeated the procedure 100,000 times for each dog. To determine the significance of each human facial expression pair at the group level, we compared the mean classifier accuracy with the classifier accuracy obtained from the permutations. In Figure 2, asterisks indicate classifier performance significantly above chance as determined by permutation testing; ∗*p* < 0.05. We compared the resulting distributions using the Kolmogorov-Smirnov test, which showed no significant difference between the distributions (*p* > 0.05).

To test whether a brain region generalized between faces expressing the same human facial expression while differentiating across human facial expressions, we conducted a Representational Similarity Analysis (RSA)^95^ for each human facial expression pair using a searchlight approach. We constructed a dissimilarity matrix (DSM) for each human facial expression pair using a single beta value for each human facial expression and run. The model represented the expected brain response where pairs of the same human facial expression were identical (dissimilarity = 0), and pairs of different human facial expressions were different (dissimilarity = 1). For each dog, we generated a correlation map by comparing the brain response with a human facial expression pair model. We selected one voxel and created a sphere around it (radius = 3 voxels) to calculate a DSM using all voxels within the sphere by computing the correlation distance between the beta values of these voxels. We calculated Spearman’s correlation coefficient between the DSM of the sphere and the human facial expression-pair model and assigned the result to the correlation map in the same coordinates as the center of the sphere. This process was repeated for all the voxels within the brain. We then averaged the correlation maps of all dogs to create a single-group correlation map for each human facial expression-pair model.

To calculate the expected group-average correlation, we used permutation testing. We repeated the process described above, but this time randomizing the human facial expression labels on each run, calculated correlation maps for each dog, and then a group correlation map. We repeated this process 100,000 times. We created a z-map by transforming each voxel of the original group correlation map to z-score and compared its value with that of the permuted maps by calculating the expected average correlation and standard deviation for that voxel. To assess which brain regions were significant, we thresholded the z-map (z > 3.1) corrected for multiple comparisons and calculated an expected cluster size distribution using the same threshold and the permuted maps. Given this cluster size distribution, we retained only clusters with sizes that were unlikely to occur by chance (*p* < 0.05).

## Supplemental information

Document S1. Figure S1

Video abstract
