# Supplementary material for: Dog brain representations of human facial expressions: Encoding happiness and differentiating specific negative expressions
Source: iScience. 2026 Aug 10;29(8):116900. doi: 10.1016/j.isci.2026.116900 (PMC13523845; doi:10.1016/j.isci.2026.116900)
Supplement: Supplementary file 1 [file mmc1.pdf]

iScience, Volume *29*

Supplemental information

Dog brain representations of human facial expressions: Encoding happiness and differentiating specific negative expressions

Raúl Hernández-Pérez, Luis Concha, Attila Andics, Rodolfo Bernal-Gamboa, and Laura V. Cuaya

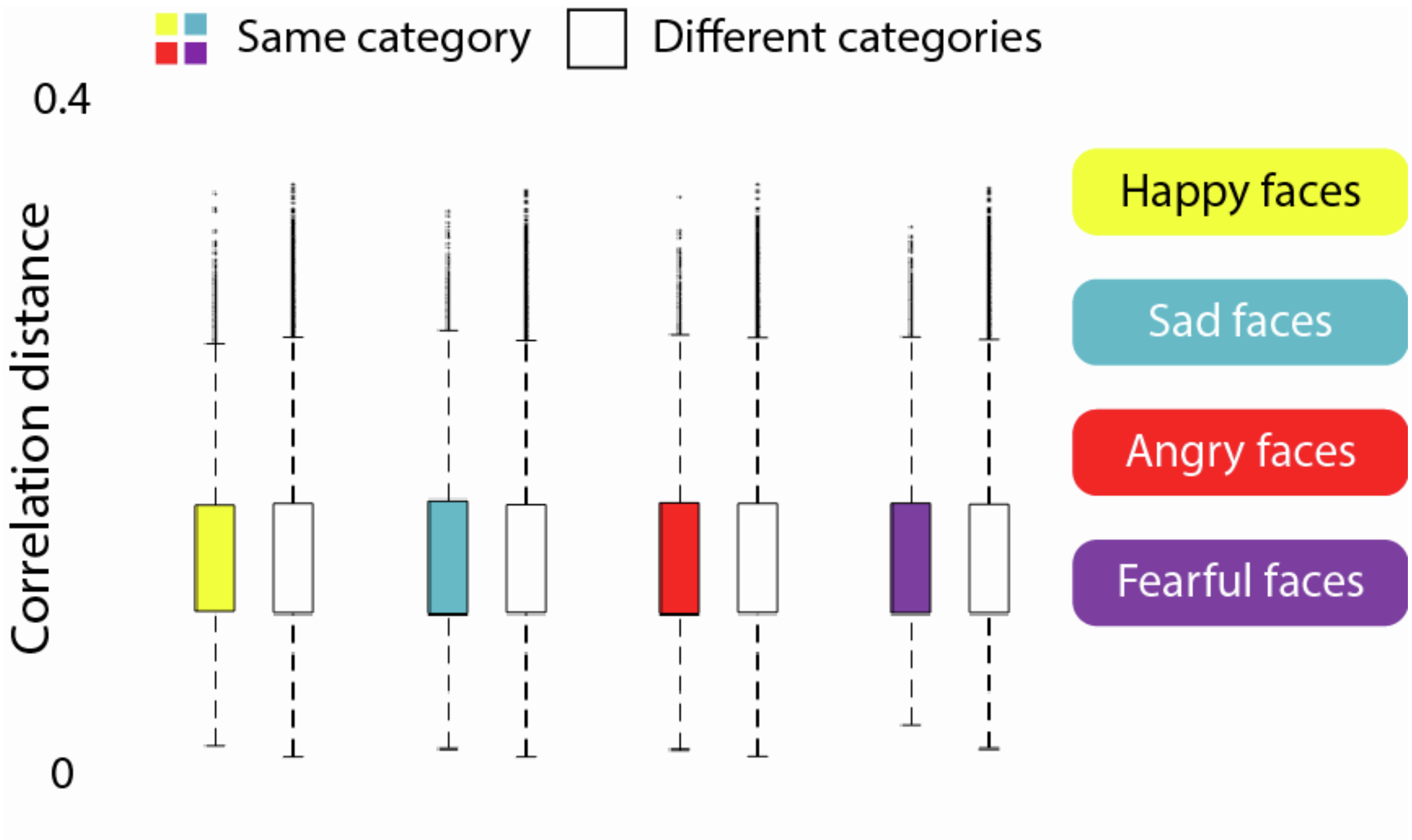

Figure S1. Within- and between-group visual-shape dissimilarity comparison of the human facial expressions presented in Experiment 2. The figure represents the correlation distance (1 – Pearson correlation) between the output of the last layer of C1 units of the HMAX model[S1] of within-group (color) and between-group (white) image pairs.

Supplemental Reference

S1. Serre, T., Wolf, L., Bileschi, S., Riesenhuber, M., and Poggio, T. (2007). Robust object recognition with cortex-like mechanisms. IEEE Transactions on Pattern Analysis and Machine Intelligence 29, 411–426.
